# Mitigation of salinity stress in tomato using green-synthesized silver nanoparticles derived from *Teucrium stocksianum*

**DOI:** 10.1186/s12870-026-08462-5

**Published:** 2026-04-25

**Authors:** Soheila Aghaei Dargiri, Davood Samsampour

**Affiliations:** Horticulture Sciences Department, Faculty of Agriculture and Natural Resource, University of Hormozgan, Bandar Abbas, Hormozgan Iran

**Keywords:** Silver nanoparticles, Teucrium stocksianum, Salinity stress, Green nanotechnology, Tomato

## Abstract

Salinity is a critical stress that severely limits tomato (*Solanum lycopersicum*) seed germination and early seedling growth. Green-synthesized silver nanoparticles (AgNPs) have emerged as a promising strategy to enhance plant tolerance to such abiotic stresses by modulating physiological and biochemical processes. In this study, a factorial experiment in a completely randomized design was conducted to evaluate the effects of green-synthesized AgNPs derived from *Teucrium stocksianum* at 0, 20, 40, and 60 ppm on tomato germination and early seedling growth under different salinity levels (0, 50, 100, and 150 mM NaCl). AgNPs were characterized using FESEM, FTIR, and XRD, confirming their crystalline structure and successful plant-mediated synthesis. Antioxidant activity measured via DPPH and ABTS assays increased with concentration, reaching 50–100% inhibition. At 150 mM NaCl, AgNPs at 20 ppm enhanced seed germination by 93.33%, accelerated germination rate, reduced mean germination time, and significantly increased stem length (85.2%), root length (125%), and seedling biomass compared to the control .These findings demonstrate that green-synthesized AgNPs provide a novel, environmentally friendly approach to mitigate salinity stress and enhance germination and early seedling growth of tomato under controlled conditions.

## Introduction

*Teucrium stocksianum* (known locally as Kalpura) is a herbal medicine that contains significant quantities of phenolic acids, flavonoids, terpenoids, and many additional redox-active compounds, all of which possess potent antioxidant, anti-inflammatory, and reducing ability [1]. The presence of these phytochemicals makes the extract a very effective natural reducer and stabiliser in the development of silver nanoparticles (AgNPs) through the use of green chemistry. In addition to donating electrons to reduce Ag^+^ ions, the bioactive compounds in the extract also stabilize and coat the silver nanoparticles with a layer of phytochemicals. This phytochemical coating provides greater stability to the nanoparticles (NPs), while enhancing their biological activity and ability to interact with plant tissues [2, 3].

AgNPs produced using green synthetic methods offer several advantages over conventional metal nanoparticles, including environmental compatibility, biocompatibility, and precise interactions with biological systems [4]. In agriculture, nanoparticles are increasingly applied due to their small size, high surface area, and ability to deliver bioactive molecules efficiently, even at low concentrations [5–8]. AgNPs may enhance seed germination and early seedling growth by improving water uptake, stimulating cell elongation, and supporting early metabolic activity, thereby helping seedlings tolerate salinity stress [9].

Salinity stress is one of the most destructive abiotic factors in tomato seedling germination and growth. The negative effects of salinity cause a decrease in free radical levels and damage to plant cells, altering their structural integrity and increasing the loss of cellular integrity, which affects their ability to grow normally as photosynthetic organisms [10]. Salinity stress causes significant reduction in plant yield and quality [11, 12]. Recent research indicates that the potential use of environmentally preferable methods to produce AgNPs may provide some mitigation to the stress of salinity, as AgNPs may affect several different molecular pathways [13, 14]. Despite the growing interest in nanoparticle applications for crop stress tolerance, data on the effects of *T. stocksianum*-derived AgNPs on tomato under salinity stress remain limited, representing a clear research gap. *T. stocksianum* was chosen due to its rich bioactive compounds, which facilitate the green synthesis of environmentally friendly nanoparticles [15–17].

In this study, we hypothesized that green-synthesized AgNPs from *T. stocksianum* can improve germination and early seedling growth of tomato under salinity stress. To test this, AgNPs were synthesized using *T. stocksianum* extracts via a green method and characterized using FESEM, FTIR, and XRD. Their effects on antioxidant activity and seedling performance under graded salinity were systematically evaluated. This work introduces these AgNPs as sustainable, biocompatible tools for mitigating abiotic stress in tomato, addressing a specific knowledge gap in the application of plant-mediated nanoparticles for horticultural crops.

## Materials and methods

### Sampling site and species verification of *T. stocksianum*

The growing area of *Teucrium stocksianum* in Isin village, Hormozgan Province, Iran, was recorded at 27°23′13.7″N, 56°40′37.0″E. The species identification and distribution were confirmed using primary sources [18, 19], Flora Iranica, and examination of herbarium specimens. The plant was officially verified by botanist Dr. Soltanipour, and a voucher specimen was deposited at the Hormozgan Province Agricultural and Natural Resources Research and Education Center Herbarium (HPANRREC) with the accession number 4540.

### Preparation of plant extract

The leaves of the plant were subjected to desiccation under ambient conditions and in the absence of direct sunlight for a duration of 14 days. The initial step involved the grinding of the substance into a fine powder. Subsequently, a quantity of 10 g of said powder, derived from the leaves, was combined with 100 ml of deionized water and subjected to boiling for a duration of 5 to 10 min. Following this, the mixture was allowed to cool down to ambient temperature. Once the plant extract was extracted, it was centrifuged twice at 10,000 rpm after being filtered through Whatman filter paper. Ultimately, the extract was ready for the production of AgNPs [20].

### Synthesis of AgNPs using *T. stocksianum* extract

AgNPs were synthesized using *T. stocksianum* leaf extract in a green synthesis approach. Three replicates were prepared using 0.25, 0.5, and 1 g of dry leaf extract dissolved in 10 mL deionized water, which was then mixed with 90 mL of 10 mM AgNO₃ solution (prepared by dissolving 1.7 g AgNO₃ in 1 L deionized water). The reaction mixture was stirred continuously at 200 rpm at room temperature (~ 25 °C) under ambient light for 90 min, during which the formation of AgNPs was monitored via a gradual color change from pale yellow to brown. The reaction was terminated once the color stabilized, indicating optimal nanoparticle formation. The nanoparticles were purified by centrifugation at 12,000 × g for 15 min, washed three times with deionized water, and redispersed to a final stock concentration of 1 mg/mL for subsequent characterization and application [20].

### Field Emission-Scanning Electron Microscopy (FE-SEM)

The morphology and particle size distribution of AgNPs were analyzed using FE-SEM. A small portion of AgNPs was placed on carbon conductive tape mounted on an aluminum stub and coated with a thin layer of gold to improve conductivity. Images were obtained under high vacuum at multiple magnifications. Particle sizes were measured from at least 200 individual nanoparticles using ImageJ software, resulting in a size range of 10–55 nm, with a mean size of 28 ± 8 nm [21].

### Fourier Transform infrared (FT-IR) spectroscopy

FT-IR analysis was performed on potassium bromide pellets prepared from the leaf extract and synthesized AgNPs (FT-IR Spectrometer, Perkin Elmer, Spectrum 100) in the 4000–280 cm⁻¹ range. This allowed identification of functional groups involved in the reduction and stabilization of AgNPs. Comparative analysis confirmed the presence of characteristic bonds responsible for bioreduction and capping of nanoparticles [22].

### XRD analysis

The crystalline structure of the biosynthesized AgNPs was examined using XRD with Cu Kα radiation (λ = 1.5406 Å) at 2θ = 20°–80°. The average crystallite size was calculated using the Debye–Scherrer equation as 27 ± 5 nm, consistent with FE-SEM measurements.

### Assessment of antioxidant activities

#### ABTS assay

The ABTS (2,2′-Azino-bis(3-ethylbenzothiazoline-6-sulfonic acid) radical cation solution was prepared and incubated until it reached an absorbance of 0.7 at 743 nm. After incubation, the solution was diluted with phosphate-buffered saline, and 100 µL of the test sample (AgNPs) at concentrations of 0.031, 0.062, 0.125, 0.25 and 0.5 mg/ml was added. The mixture was allowed to react for 6 min at room temperature. Trolox was used as a standard to construct the calibration curve. Absorbance was measured at 734 nm using a spectrophotometer. The ABTS radical scavenging activity (RSA%) was calculated using:

$$\mathrm{RSA}\;(\%)\;=\;((_{s}A_{c}-\;A)\;/\;_{c}A)\;\times\;100$$

where _c_*A* is the absorbance of the ABTS solution without sample (control), and _s_*A*​ is the absorbance with the test sample. All measurements were performed in triplicate, and results are expressed as mean ± standard deviation.

### DPPH assay

Free radical scavenging activity was evaluated using 1,1-diphenyl-2-picryl-hydrazyl (DPPH). A 0.1 mM DPPH solution in methanol was prepared. 3 mL of each test sample AgNPs at concentrations of 0.031, 0.062, 0.125, 0.25 and 0.5 mg/ml was mixed with 2 mL of DPPH solution. The mixture was incubated in the dark at room temperature for 30 min. Ascorbic acid was used as a standard for calibration. Absorbance was measured at 517 nm, and the percentage of scavenging activity was calculated using the formula [23].

$$\mathrm{RSA}\;(\%)\;=\;((_{s}A_{c}-\;A)\;/\;_{c}A)\;\times\;100$$

where _c_*A*​ is the absorbance of the DPPH solution without sample (control), and _s_*A* ​ is the absorbance of the DPPH solution with the test sample. All assays were performed in triplicate, and results are reported as mean ± standard deviation.

### In vitro: tomato seed germination under salt stress

The evaluation of how green-synthesis AgNPs affect the germination of tomato seeds through a Petri dish assay. Seeds were purchased from Pakán Seed Company located in Isfahan, and the type of seed used was 8320. Tomato seeds were surface-sterilized by immersion in 70% (v/v) ethanol for 30 s, followed by treatment with 0.5% (v/v) sodium hypochlorite solution for 10 min under gentle agitation. After sterilization, the seeds were rinsed thoroughly three times with sterile distilled water to remove any residual disinfectant before further experimental procedures. To assist with the removal of any contaminating material, they were washed four times with sterile distilled water, with each wash being three minutes in duration, followed by a 30-minute soak in sterile distilled water. All sterilization and washing steps were repeated three times to assure that all seeds were completely disinfected and properly distributed on the samples. Transfers and other essential processes throughout the time period were accomplished with a laminar flow hood. Subsequently, the seeds underwent soaking for around 120 min in AgNPs solutions containing various concentrations of the chemical as follows: )0, 20, 40, 60 ppm) [24]. Using the three replicates for each treatment, we placed 30 seeds into each 80 mm line petri dish containing filter paper moistened with the following NaCl (sodium chloride) solution concentrations; 0, 50, 100, 150 mM. All of the Petri dishes underwent a 16-hour cycle of light/8-hour cycle of dark photoperiods at 25 ± 1 °C. Daily records of germination were kept through 20 days. After the period of germination, caliper measurements were used to measure stem length (SL) and root length (RL). Both seedling fresh weight (SFW) and dry weight (SDW) were determined using a very high-quality scale accurate to 0.0001. The standard calculations for germination metrics, which include germination percentage (GP), mean germination time (MGT), mean germination rate (R), and minimum daily germination (MDG), were followed [25–27].

### Data analysis

The experiment was conducted as a factorial arrangement (AgNPs concentration × NaCl level) in a completely randomized design (CRD). Each Petri dish, containing 30 seeds, was considered one experimental unit, and three independent Petri dishes were used per treatment combination. Data were analyzed using two-way ANOVA to evaluate the effects of AgNPs concentration, salinity level, and their interaction on the measured parameters. SAS (Version 9.4) was used to compare the means and analyse the LSD test at a significant level (*p* ≤ 0.05). Principal component analysis (PCA) was conducted using XLSTAT version 2020 (www.xlstat.com, Addinsoft SARL). Hierarchical cluster analysis and Pearson correlation were performed using R software (www.r-project.org).

## Results and discussion

### FESEM analysis of NPs morphology

**Fig. 1:**
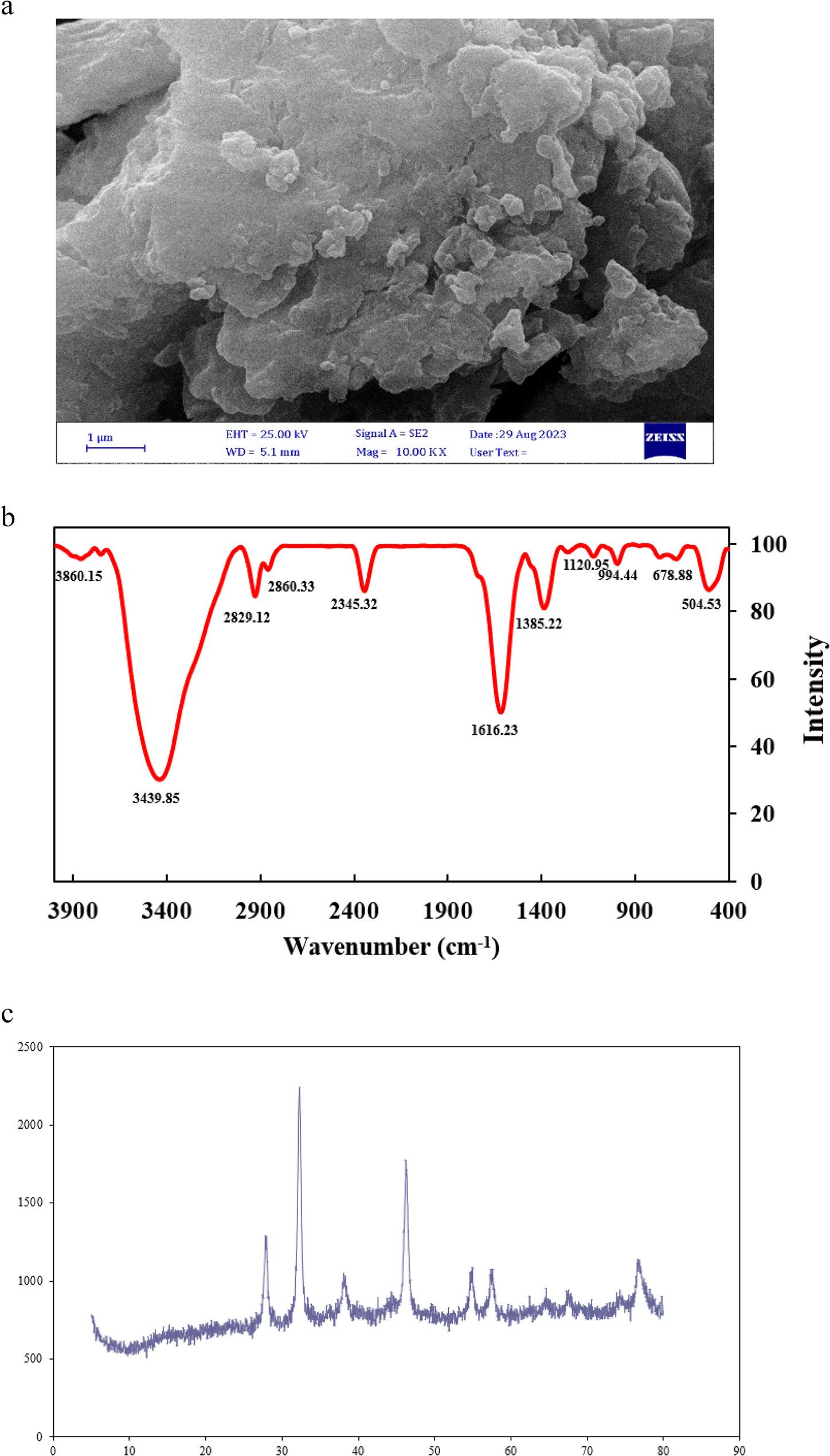
**a** FE-SEM (**b**) FTIR and (**c**) XRD image of silver nanoparticle showing the distribution of sizes of the silver nanoparticles

FESEM analysis at 10,000× magnification (scale bar: 1 μm) revealed that the AgNPs possessed a rough and irregular morphology. The synthesized AgNPs exhibited diameters ranging from 55.80 to 75.92 nm (Fig. 1a). SEM analysis suggests that diverse phytochemicals including alcohols, phenols, aldehydes, esters, amines, and carboxylic acids present on the surface of the nanoparticles are responsible for the reduction and stabilization of the synthesized nanomaterials [28–30]. SEM analysis has consistently shown that the nanoparticles produced are predominantly spherical, with sizes ranging from about 10–20 nm [31] to 25–40 nm [32], and up to 36–97 nm depending on the plant species used [33].

### FTIR profile of biosynthesized AgNPs

The FTIR spectrum of the synthesized nanoparticles exhibited a prominent broad band at 3439.85 cm⁻¹ corresponding to O–H stretching vibrations, along with a distinct peak at 1616.23 cm⁻¹ attributed to C = O or aromatic C = C stretching (Fig. 1b). FTIR spectra typically reveal the presence of functional groups like phenols, amines, and carboxyls, which are responsible for the bioreduction and stabilization of AgNPs [34, 35]. The chemical heterogeneity and shifts observed in FTIR spectra further indicate changes in the chemical environment due to nanoparticle formation [36]. In study *Eucalyptus globulus* yields smaller AgNPs (17.5 ± 5.89 nm) compared to *Salvia officinalis* (34.3 ± 7.76 nm) [34]. Another recent study demonstrated significant heterogeneity in chemical shifts among functional groups in AgNPs synthesized from *Enantia chlorantha* extracts, indicating changes in chemical surroundings and supporting the use of FTIR for detailed characterization of plant-based nanomaterials [36].

### XRD confirmation of AgNPs crystallinity

Characteristic diffraction peaks confirmed the formation of pure, face-centered cubic silver (JCPDS No. 04-0783). The peaks at around 30°, 45°, and 50° (2θ), correspond to characteristic lattice planes and represent a successfully formed crystalline NPs structure. The absence of secondary or extra peaks indicates that the NPs are of high purity, whereas the broadening of some of the peaks constitutes evidence that nano-particles were formed (Fig. 1c). XRD analysis consistently confirms the crystalline nature of AgNPs synthesized from various plant extracts, with crystallite sizes typically ranging from 10 to 33 nm depending on the plant source and synthesis conditions [37, 38]. AgNPs synthesized using Rubus ellipticus root extract exhibited a crystalline structure with sizes ranging from 13.85 to 34 nm, confirmed by XRD [39]. Similarly, extracts from Berberis vulgaris, Brassica nigra, and Lavandula angustifolia produced AgNPs with bimodal size distributions and enhanced antimicrobial activity, with XRD confirming the presence of metallic silver (Ag_0_) [37]. A study using *Citrus aurantifolia* fruit peel extract was presented. XRD analysis confirmed the crystalline structure of the silver nanoparticles and revealed diffraction peaks at 2θ values ​​of 32.36°, 38.24°, 46.26° and 57.44°, respectively, corresponding to the (122), (111), (200) and (241) planes [40].

### DPPH radical scavenging activity of synthesized NPs

**Fig. 2:**
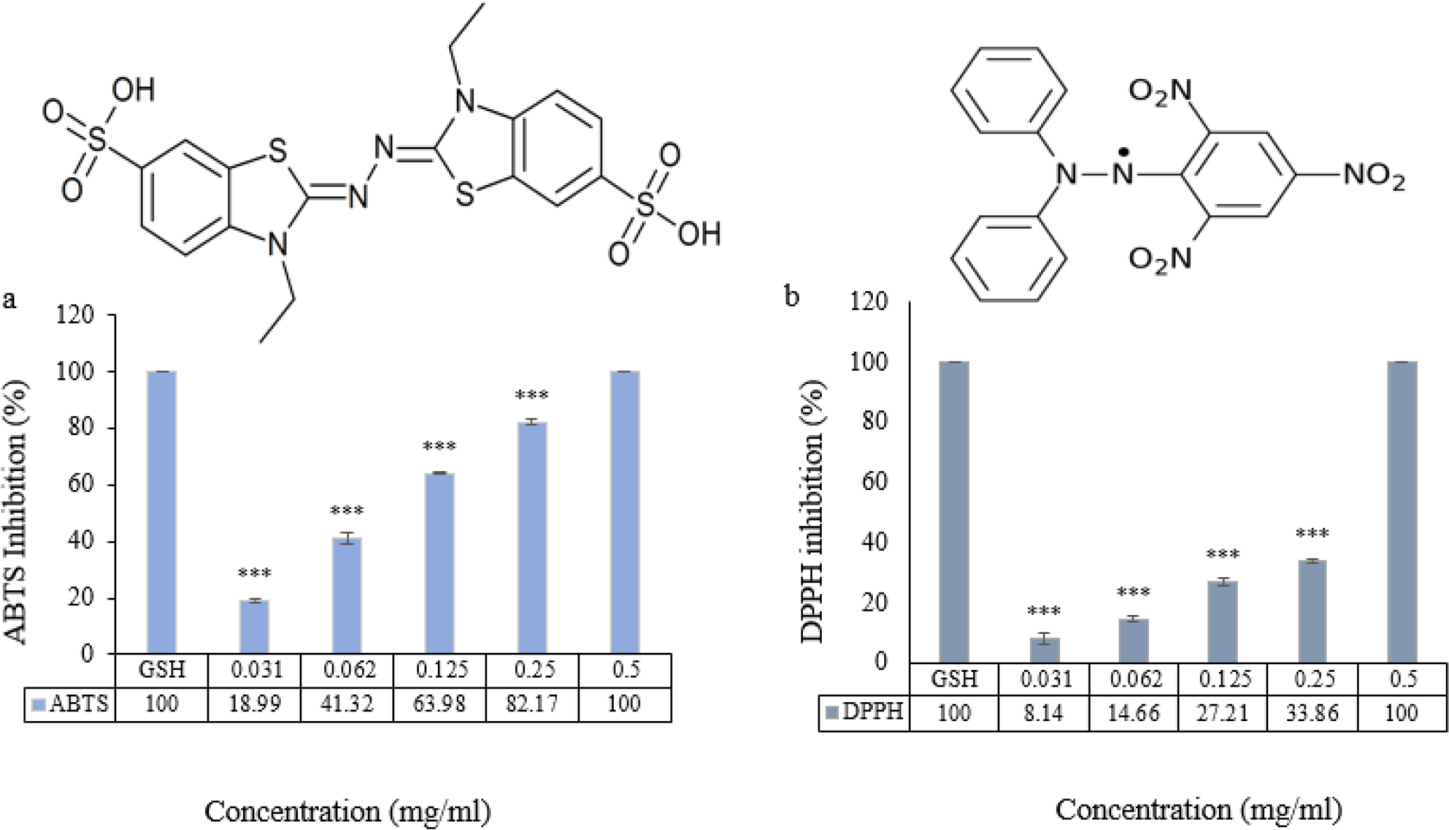
**a** ABTS (**a**) and DPPH (**b**) radical scavenging activity of the synthesized nanoparticles. Data are presented as mean ± SE (*n* = 3). Asterisks (^*^) indicate significant differences between treatments based on the LSD test (*p* ≤ 0.05)

AgNPs synthesized from *T. stocksianum* extract were evaluated for their antioxidant activity using DPPH and ABTS assays (Fig. 2). The results showed that at a concentration of 0.25 mg/ml, ABTS and DPPH inhibition decreased significantly compared to the control, showing 17.83% and 14.66% inhibition, respectively (*p* ≤ 0.05). In general, higher concentrations of AgNPs enhanced radical scavenging activity, although their antioxidant potential remained lower than that of GSH, the standard reference (Fig. 2a, b). The antioxidant potential of these nanoparticles is attributed to the presence of phytoconstituents like phenolic compounds, which enhance their radical scavenging abilities [41]. Numerous investigations conducted by Khan et al. [42] using DPPH, ABTS^+^, and H_2_O_2_ radical scavenging methods have revealed that AgNPs manufactured using the *Phytolacca octandra* herb extract biosynthetic method exhibit a substantially higher level of antioxidant properties than did the methanol extract of *P. octandra* alone. The results of the investigation showed that, at the maximum prepared concentration of 250 µg ml, the newly created AgNPs had higher ABTS radical scavenging activity (77%) and DPPH radical scavenging activity (87%), in comparison to the *L. royleana* leaf extract (62%) [43].

### AgNPs enhances seed germination under salinity stress

**Fig. 3:**
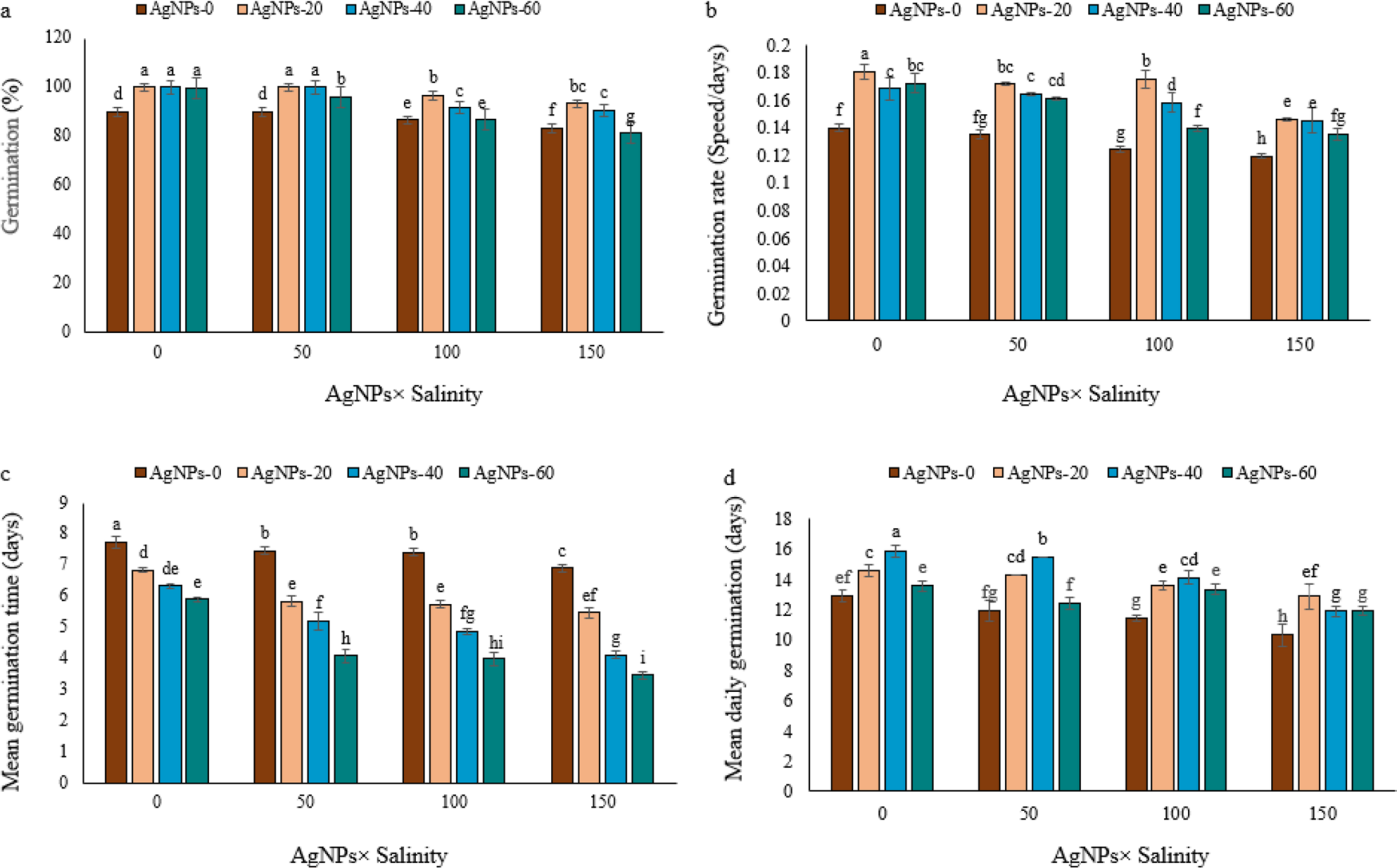
Effect of silver nanoparticles (AgNPs) (0, 20, 40, 60 ppm) on tomato germination parameters under salinity stress (0, 50, 100, 150 mM NaCl) (**a**) germination percentage, (**b**) germination rate, (**c**) mean germination time, (**d**) mean daily germination. Data represent mean ± SE (*n* = 3). Different letters indicate significant differences between treatments based on LSD test (*p* ≤ 0.05)

The interaction of 20 ppm AgNPs and 150 mM salinity stress increased GP by 93.33% compared to the control (*p* ≤ 0.05) (Fig. 3a). AgNPs can help increase the permeability of cell membranes and facilitate the transport of water and nutrients into the plant, which helps maintain the plant’s water and nutritional balance in saline conditions [44]. A study showed that pretreatment of spinach seeds with green AgNPs at optimal concentrations may increase their tolerance to salt stress and potentially improve germination and growth under saline conditions [45]. In order to evaluate the effects of biosynthesized AgNps on *Cucumis sativus* L. seedlings, the results showed that both forms of silver significantly reduced growth at higher concentrations, which may be due to increased silver accumulation in plants (4708.2 ± 108.75 mg/kg) [46]. Darvishzadeh et al. [47] conducted a study on *C. sativus* and reported that the application of AgNPs at a concentration of 40 ppm significantly increased the germination percentage of cucumber seeds under saline stress.

### AgNPs accelerates tomato seed germination rate under salinity stress

The highest R was observed in the 20 ppm AgNPs treatment at 0.180 per day at 150 mM salinity (*p* ≤ 0.05) (Fig. 3b). AgNPs, with their antioxidant properties, can reduce the production of free radicals and toxic peroxides caused by salt stress in plants. This reduction in oxidative stress limits damage to cell membranes and other plant organs, and maintains cell integrity during the germination stage [48]. In one study, pretreatment of tomato seeds with biosynthesized silver nanoparticles derived from the leaf extract of *Larrea tridentata* (4–26 nm) at a concentration of 4.03 mg l^− 1^ increased seed germination to approximately 90%, compared with less than 60% in the control [49]. Although direct studies on AgNPs in tomato are limited, research on biogenic AgNPs in other plant species, such as *Echinops macrochaetus*, shows that AgNPs can ameliorate salinity stress and improve growth parameters when used as seed priming agents [50].

### AgNPs reduces tomato seed germination time under salinity

MGT decreased with increasing concentrations of AgNPs across all salinity levels. At 20 ppm AgNPs, MGT was reduced to approximately 5.48 days under 150 mM NaCl. A further decline was observed at 40 and 60 ppm, where MGT reached 4.12 and 3.47 days, respectively, at 150 mM NaCl (*p* ≤ 0.05) (Fig. 3c). The faster germination time at higher concentrations of AgNPs is likely related to increased water uptake, thus enhancing metabolic processes and allowing for faster germination and seedling establishment cycle [51]. Green silver nanoparticles synthesized using Hibiscus sabdariffa leaf extract show that although salt stress significantly reduces seed germination and growth parameters, the use of green silver nanoparticles can reduce these adverse effects [52]. Parveen and Rao [53] and Rezvani et al. [54] conducted studies on the effects of AgNPs on seed germination in *Pennisetum* and saffron (*Crocus sativus*), respectively. They concluded that AgNPs concentrations ranging from 20 to 60 ppm had significant effects on germination parameters in both plant species.

### AgNPs effects on daily germination under salinity

The 20 ppm AgNPs treatment showed similar results to the control with a slight decrease from 12.86 days to 12.06 days at 150 mM salinity stress in MDG. The MDG reached its lowest level, 11.9 days, at 150 mM salinity stress when 60 ppm was used (*p* ≤ 0.05) (Fig. 3d). AgNPs enhance seed germination under salt stress by regulating physiological functions such as nitrogen metabolism and water uptake during seed germination [52]. Studies have shown that when silver nanoparticles are added at lower doses (onion: 25 ppm; tomato: 4.03 mg l^− 1^), tomato seed germination can reach 90%, while optimal onion seed growth can be found at 25 ppm of AgNPs [55, 56]. According to Mahakham et al. [57] 20 ppm silver nanoparticles have been shown to increase germination, growth rate and vigor of *Lactuca sativa* and *Oryza sativa* seeds.

### AgNPs -driven enhancement of shoot and root growth under salinity stress

**Fig. 4:**
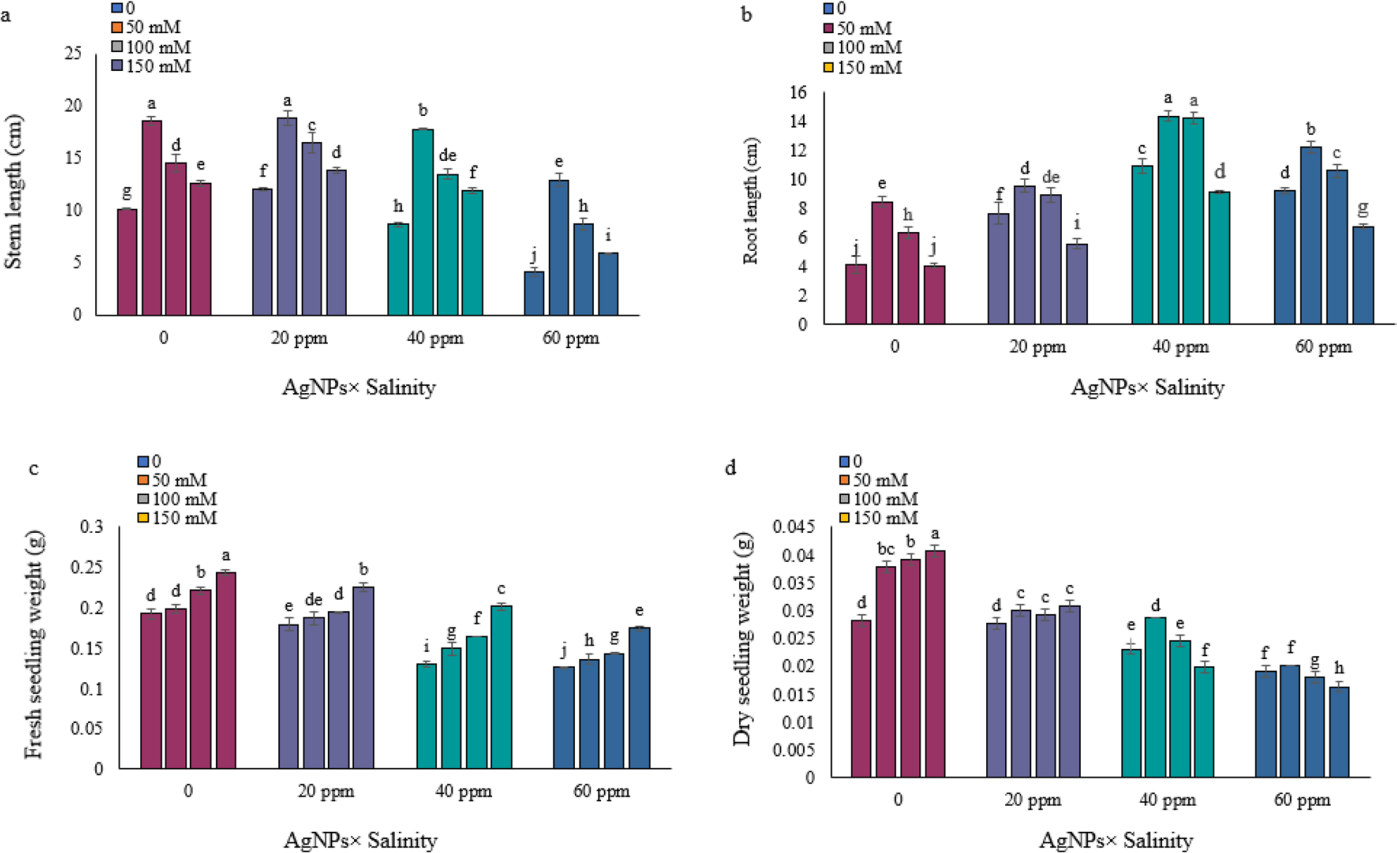
Effect of silver nanoparticles (0, 20, 40, 60 ppm) on tomato growth parameters under salinity stress (0, 50, 100, 150 mM NaCl) (**a**) stem length, (**b**) root length, (**c**) fresh seedling weight, (**d**) dry seedling weight. Data represent mean ± SE (*n* = 3). Different letters indicate significant differences between treatments based on LSD test (*p* ≤ 0.05)

Based on the results, 20 ppm treatment increased the SL by 85.218% compared to the control at 150 mM salinity stress (*p* ≤ 0.05) (Fig. 4a). The 40 ppm AgNPs treatment increased the measured parameter by 69.82% and 125.19% compared with the control at 50 and 100 mM salinity, respectively; however, a decrease was observed at 150 mM salinity (*p* ≤ 0.05) (Fig. 4b). Under salinity stress, the application of AgNPs may enhance plant growth by modulating ionic balance and improving stress signaling pathways [58]. According to the findings presented by Kurt and Ateş [59] the use of AgNPs has a positive impact on morphological characteristics of boysenberry (*Rubus ursinus*) by maintaining water balance and promoting growth rate in saline conditions. Studies conducted by Sadat-Hosseini et al. [52] have revealed that AgNPs derived from the extraction of *Hibiscus sabdariffa* leaves have the capacity to reduce some of the adverse effects associated with salinity in regard to germination and early growth of seeds such as height, weight and biomass.

### Seedling biomass response to AgNPs under severe salinity

The treatment with 20 ppm AgNPs at 150 mM salinity stress increased the fresh and dry weights by 25.69% and 11.59%, respectively, compared to the control (*p* ≤ 0.05) (Fig. 4c, d). AgNPs can stimulate cell division and differentiation in roots and stems, which leads to an increase in cell number and size, and ultimately increases the fresh and dry weight of the seedling [60]. For example, colloidal AgNPs (100 ppm) applied to ‘Bright Pixi’ lily plants under NaCl stress (600 mM) offset reductions in fresh bulb weight, bulb diameter, and the number of scales in bulbs, effectively improving bulb yield and growth despite salinity stress [61]. Similarly, green-synthesized AgNPs using *H. sabdariffa* leaf extract were shown to alleviate the negative impact of salinity on seed germination and growth parameters, including fresh and dry weights of leaves and roots, although salt stress still caused significant reductions in these metrics [52]. In quinoa, the addition of chemically synthesized AgNPs (25, 50, and 75 mg l^-1^) to growth media was tested to boost salt stress mitigation, but all growth parameters, including fresh and dry weight, were negatively affected by NaCl at all levels [62].

### Correlation and principal component analysis

**Fig. 5:**
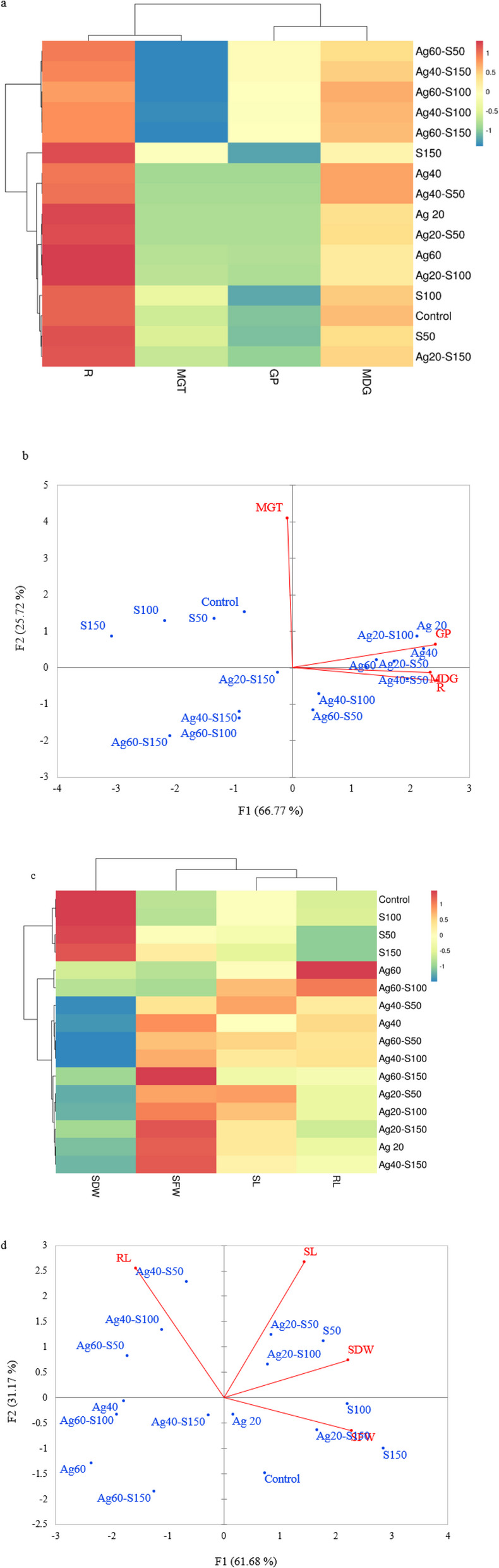
**a-c** Heatmap showing significant positive correlations between germination parameters and AgNPs treatments at 0, 20, 40, and 60 ppm under different salinity levels (0, 50, 100, and 150 mM) including non-saline conditions. Hierarchical clustering analysis (HCA) of Pearson’s correlation coefficient (r) values of variable traits, where the color scale that indicates r coefficient values (*r* = 1 to -1) indicates positive (red) and negative (blue) correlations (**b-d**) Principal component analysis (PCA) biplot illustrating relationships between germination parameters and AgNPs treatments at various concentrations (0, 20, 40, and 60 ppm) under varying salinity level

According to heat map analysis, the relationship between germination traits and silver nanoparticles during different salinity treatments due to the application of AgNPs on seeds was significant (*p* ≤ 0.05). A positive relationship of R rate was observed between silver nanoparticles compared to untreated controls and silver nanoparticles with a concentration of 20 ppm at salt concentrations of 100–150 mM. The MDG was positively and significantly correlated at 40 ppm AgNPs concentration (non-saline conditions), indicating that. Furthermore, at 100 and 150 mM salinity, AgNPs were positively correlated with MDG at concentrations of 40 and 60 ppm, respectively (Fig. 5a).

Two significant components of the principal component analysis (PCA) explained 92.49% of the variance of the data, with PC1 accounting for 66.77% of the variance and PC2 accounting for 25.72%. A positive correlation was observed between GP percentage and Ag AgNPs NP treatments with concentrations of 20 and 60 ppm (non-saline conditions) and 20 and 40 ppm AgNPs in 50 and 100 mM salinity conditions. MDG and R showed significant similarity in the 40 ppm AgNPs treatment under 50 mM salinity stress. On the contrary, MGT showed positive similarity with increasing salinity levels in all treatments (Fig. 5b).

The analysis showed significant correlation of RL with 60 ppm AgNPs in both non-saline and 100 mM salinity conditions. SL also showed a positive correlation with 20 ppm AgNPs at 50 and 100 mM salinity and with 40 ppm at 50 mM salinity. In addition, SFW showed a significant correlation with AgNPs at concentrations of 20, 40 and 60 ppm at high salinity (150 mM) and also with 20 ppm under non-saline conditions. SDW showed a variable correlation pattern that was influenced by salinity intensity and AgNPs treatments (Fig. 5c).

Principal component analysis revealed that two components together accounted for 92.85% of the total variance. Component 1 (PC1) accounted for 61.68%, while component 2 (PC2) accounted for 31.17% of the total variance. Biplot analysis of PCA indicated positive relationships between SDW and 20 ppm AgNPs treatment at salinity levels of 50 and 100 mM. SFW also showed a significant positive correlation with AgNPs treatments under 150 mM salinity. In addition, RL was positively correlated with 40 ppm AgNPs at 50 mM salinity (Fig. 5d).

## Conclusion

Green-synthesized silver nanoparticles (AgNPs) from *Teucrium stocksianum* improved tomato (*Solanum lycopersicum*) seed germination and early seedling growth under salinity stress. At 20 ppm, AgNPs enhanced germination percentage, accelerated germination rate, reduced mean germination time, and increased stem and root growth as well as seedling biomass. These results indicate that AgNPs may help mitigate salinity-induced inhibition during the early growth stage. However, the study was limited to Petri-dish conditions, and long-term performance, physiological mechanisms, and environmental impacts were not assessed. Future research should include field trials, molecular validation, dose–response safety studies, and environmental risk assessments to support practical agricultural applications.

## Data Availability

All data generated or analysed during this study are included in this manuscript.
